# Organ preservation in T4a laryngeal cancer: is transoral laser microsurgery an option?

**DOI:** 10.1007/s00405-013-2382-7

**Published:** 2013-02-14

**Authors:** Martin Canis, Friedrich Ihler, Alexios Martin, Hendrik A. Wolff, Christoph Matthias, Wolfgang Steiner

**Affiliations:** 1 Department of Otorhinolaryngology, Head and Neck Surgery, University of Göttingen, Robert-Koch-Str. 40, 37099 Göttingen, Germany; 2 Department of Audiology and Phoniatrics, University of Berlin, Berlin, Germany; 3 Department of Radiation Oncology, University of Göttingen, Göttingen, Germany

**Keywords:** T4 laryngeal carcinoma, Transoral laser microsurgery, Oncologic results, Functional outcomes

## Abstract

The main objective of this study is to assess the feasibility of transoral laser microsurgery (TLM) in the treatment of T4a laryngeal cancer and to report the oncological and functional outcomes. This is a retrospective case-series study, held in a single-institute, academic tertiary referral center. Seventy-nine patients with previously untreated T4a glottic (*n* = 31, 39 %) or supraglottic laryngeal carcinoma (*n* = 48, 61 %) were included in this study. Five patients (6 %) were treated exclusively by TLM, 16 (20 %) had TLM and unilateral neck dissection, 27 (35 %) had TLM and bilateral neck dissection. Adjuvant (chemo)radiotherapy was additionally administered in 26 (33 %) cases following TLM and neck dissection, and in 5 (6 %) cases after TLM without neck dissection. The main outcome measures included organ preservation, local control, functional outcome, overall, recurrence-free, and disease-specific survival. The median follow-up period was 49 months, 5 year organ preservation rate and local control rate were 80.0 and 67.2 %, 5 year overall, recurrence-free and disease-specific survival were 55.8, 61.9 and 71.8 %. The 5 year overall survival rates were 62.5 % in pN0 cases and 57.2 % in cases with pN-positive neck disease. With respect to survival, these results are comparable to total laryngectomy, while being superior to primary (chemo)radiotherapy. TLM results in a low morbidity, rapid recovery and good function and can be a valid option for organ preserving surgery of pT4a glottic and supraglottic cancer.

## Introduction

Malignant tumors of the head and neck represent 6.6 % of all tumors registered by the American Cancer Society, with laryngeal cancer accounting for almost 21 % [1]. While the incidence and mortality of laryngeal cancer seems to decline [2], no significant improvements regarding overall survival of advanced laryngeal cancer have been achieved in the last three decades.

After the introduction of the CO_2_ laser for the treatment of benign and very early malignant laryngeal lesions by Jako and Strong [3], Steiner expanded the indications of transoral laser microsurgery (TLM) to all tumor categories of the upper aero-digestive tract [4–7] in the early 1980s.

Using TLM, it is possible to minimize the loss of healthy tissue, to avoid extensive reconstruction procedures, and primary tracheotomies in most cases and to lower the overall morbidity. This method can be seamlessly integrated into any therapeutic regimen while maintaining all salvage treatment options. Prerequisites for using this TLM are a detailed knowledge of endoscopic anatomy, microsurgery and proficiency in the use of lasers.

The aim of the present study was to evaluate the oncologic and functional outcome of TLM for T4a glottic and supraglottic cancer and to prove the validity of this treatment method by comparing it to total laryngectomy or primary (chemo)radiotherapy as current standard of care.

## Patients and methods

Between August 1987 and December 2006, a total of 79 patients with pT4a glottic and supraglottic laryngeal squamous cell carcinoma were treated with curative intent at the Department for Otorhinolaryngology—Head and Neck Surgery, University of Göttingen.

Previously untreated tumors were staged according to the current classification of the Union for International Cancer Control (UICC) and the American Joint Committee on Cancer (AJCC) [8]. We included moderately advanced tumors that invaded through the thyroid cartilage and/or soft tissues of the neck. Exclusion criteria for this study were: non-squamous cell carcinoma tumors, simultaneous second primary or distant metastases, and N3 neck disease. All patients were exclusively treated by TLM. Open surgery procedures were excluded. Further exclusion criteria were bilateral arytenoid fixation and deep infiltration of the neck by the primary tumor with involvement of the carotid artery.

Of 176 patients with a T4a laryngeal cancer presenting for treatment in our hospital, 97 had to be excluded. The reasons were: (a) treatment without TLM in 35 cases (open surgery in three cases, total laryngectomy in three patients, 13 cases were treated primarily by (chemo)radiotherapy and palliative treatment in 16 cases); (b) treatment with TLM but exclusion due to exclusion criteria in 62 cases (20 patients with recurrent or residual disease after primary treatment elsewhere, previous treatment for cancer in the upper aero-digestive tract (eight patients) or simultaneous second primaries (eight patients), 26 patients were excluded because of N3 neck disease, non-SCC histology, simultaneous distant metastases or death before therapy.)

A total of 79 patients fulfilled the inclusion criteria and were statistically analyzed of which, 69 male (87 %) and 10 female (13 %), 31 patients with glottic (39 %) and 48 patients with supraglottic laryngeal carcinoma (61 %). The age ranged from 32 to 80 years the median age being 57 years.

### Preoperative examination

Preoperatively, all patients underwent rigid or flexible endoscopic examination of the oral cavity, pharynx and larynx whilst awake. This was followed by sonography of the neck.

Patients initially mainly underwent CT scanning of the neck while later MRI was added when deemed necessary. Standard diagnostics included X-ray examination of the chest and sonography of the abdomen. Panendoscopy was performed under general anesthesia at the beginning of the surgical procedure with the intention to exclude any second primary tumor in the aero- and upper digestive tract followed by laser biopsy if the carcinoma was not yet proven by histology.

### Treatment of the primary tumor

The surgical procedures were performed under general anesthesia and with orotracheal intubation. Different closed or distending laryngoscopes (Karl Storz, Tuttlingen, Germany) were used for adequate exposure of the tumor. Depending on localization and size of the tumor, the endotracheal tube was placed above the upper blade of the laryngoscope if exposure of the posterior commissure was required. Occasionally, the endotracheal tube had to be removed temporarily to resect subglottic or tracheal tumor spread in apnea. As good exposure and visualization are prerequisites of a safe and successful resection, the laryngo-pharyngoscope sometimes had to be repositioned several times during the operation. To remove laser plume, the laryngo-pharyngoscopes were equipped with integrated suction tubes.

Tumors were excised in a step-wise fashion with specific blocks of tumor being consecutively removed. In contrast to the open classic en-bloc resection, the tumor was resected endoscopically in a step-by-step procedure under microscopic magnification using CO_2_ laser in continuous superpulse mode [9]. Hereby, the surgeon follows the tumor, while preserving as much as possible healthy and functionally important tissue. The specific tissue cutting characteristics of the CO_2_ laser helps the surgeon to differentiate between healthy tissue and tumor. Steiner [6] introduced this unconventional technique in the early 1980s showing that cutting through the tumor is necessary and justified, because the lymphatic vessels of the wound margins are sealed immediately, as shown by Werner et al. [10].

Tumor was resected under the microscope with surgical resection margins in healthy tissue from 2 to 3 mm (glottis) or 5 to 10 mm (supraglottis). As the quality of the postoperative function depends directly on the extent of the resection, it was our goal to preserve as much healthy laryngeal tissue as possible. If the histopathological analysis of the resected specimens by frozen sections showed a positive resection margin, an additional resection was carried out to obtain negative margins until an adequate distance between the margin of the tumor and the resection borders was achieved. However, the number of analyzed frozen sections depended on the experience of the surgeon and of the localization and extent of the tumor.

Tumors that extended to the paraglottic space required resection that reached laterally to the thyroid cartilage and caudally to the cricoid cartilage. If necessary, the arytenoid cartilage and/or parts of the thyroid or cricoid cartilage and extralaryngeal tissue were included in the endoscopic resection.

We used a CO_2_ laser system to perform the surgical procedures (40c, Lumenis, Dreieich, Germany). It was equipped with a micromanipulator attached to the operating microscope (OPMI Vario/S88, Zeiss, Göttingen, Germany). The focus diameter of the micromanipulator was 0.5 and 0.25 mm, respectively; approximately 2,080–3,900 W/cm^2^ of laser energy were transmitted. The amount of laser power used varied from 3 W for subtle resections to 15 W when cutting through a large tumor mass. This administered laser power was able to coagulate vessels with a maximum diameter of 0.5 mm. Larger vessels were coagulated with mono- or bipolar forceps. Vascular clips were applied to the superior artery and branches. If considerable portions of cartilage were exposed or partially removed during resection of the tumor, we administered antibiotics perioperatively to avoid perichondritis. Duration of surgery depended on the localization and the extent of the tumor, exposure of the surgical site and experience of the surgeon. Mean duration was 2–3 h extending up to 5 h when small closed laser laryngoscopes had to be used for better exposure of the tumor.

### Treatment of the neck

Neck dissections were mainly selective (N0–N2) and usually performed as a delayed procedure after initial TLM. Delayed neck dissection could be combined with a second microlaryngoscopic exploration if the pathology report after initial TLM indicated that this was advisable.

### Adjuvant (chemo)radiotherapy

Adjuvant (chemo)radiotherapy was performed in cases of N2 neck disease or when the histopathological examination revealed lymphangiosis carcinomatosa and/or extracapsular tumor spread. If it was impossible to prove all margins we recommended adjuvant treatment to the patients independent of neck disease. (Chemo)radiation always was administered to both the larynx and the neck. Overall 31 patients received radiation, concomitant chemotherapy was given in 11 cases.

### Statistical methods

All survival rates were calculated using the Kaplan–Meier method. The assessed endpoints were overall, recurrence-free, and disease-specific survival, local and loco-regional control, distant metastases and second primaries.

Overall survival was calculated on deaths from all possible causes, disease-specific survival on deaths from laryngeal cancer. For calculating recurrence-free survival, intercurrent deaths and deaths due to secondary primary tumors as well as patients alive without recurrences were regarded as censored observations. Events included local and regional recurrences, distant, recurrent and late metastases and deaths due to disease. For the calculation of local control rate, only local recurrences were considered as events while patients alive without local recurrence or death regardless of reason were counted as censored observations. The definition of local recurrence included carcinoma in situ as well as carcinoma occurring after completion of primary treatment. Survival times were calculated from the day of surgery to the date of the occurrence of the event or the date of the last follow-up.

## Results

### Oncologic results

Five patients (6 %) were treated exclusively by TLM, 16 (20 %) had TLM and unilateral neck dissection, 27 (35 %) had TLM and bilateral neck dissection. Adjuvant radio(chemo)therapy was administered in 26 (33 %) cases following TLM and neck dissection, and in 5 (6 %) cases after TLM without neck dissection.

Selective neck dissection (level II/III) was performed in 69 patients (87 %), 52 patients (75 %) were treated bilaterally, 17 (25 %) were treated unilaterally to the primary tumor site. In addition, level I was dissected in eight patients, level IV in six patients and level V in three patients. A bilateral modified radical neck dissection (level I–V) was done in three cases.

### Local and loco-regional control

10 of 31 (32 %) patients with pT4a glottic carcinoma developed local or loco-regional recurrence. Salvage therapy consisted of TLM with adjuvant radiotherapy (*n* = 3) or revision neck dissection followed by radiotherapy of the larynx and the neck (*n* = 2). Total laryngectomy was necessary in five cases.

Local or loco-regional recurrence was observed in 13 of 48 (27 %) patients with supraglottic cancer. Salvage therapy consisted of TLM with adjuvant radiotherapy (*n* = 2) or revision neck dissection followed by radiotherapy of the larynx and the neck (*n* = 2). Total laryngectomy was not avoidable in nine cases. rpT categories of the first local and loco-regional recurrence of 23 patients are given in Table 1. Table 2 gives patterns of TNM-related treatment failures after initial therapy.

**Table 1:** rpT categories of all 23 first local and loco-regional recurrences

rpT	No.	[%]
pT4 glottic (*n* = 31)		
1	3	10
2	2	6
3	2	6
4	3	10
	10	
pT4 supraglottic (*n* = 48)		
1	2	4
2	2	4
3	3	6
4	6	13
	13

**Table 2:** Treatment of first failures after initial therapy depending on T-category

pT4	No.	Loc. rec. no.	Locoreg. rec. No.	Late metastasis no.	Rec. metastasis no.	Σ
Glottic	31	9 (29 %)	1 (3 %)	1 (3 %)	0	11
Supraglottic	48	12 (25 %)	1 (2 %)	1 (2 %)	2 (4 %)	16

**Fig. 1:**
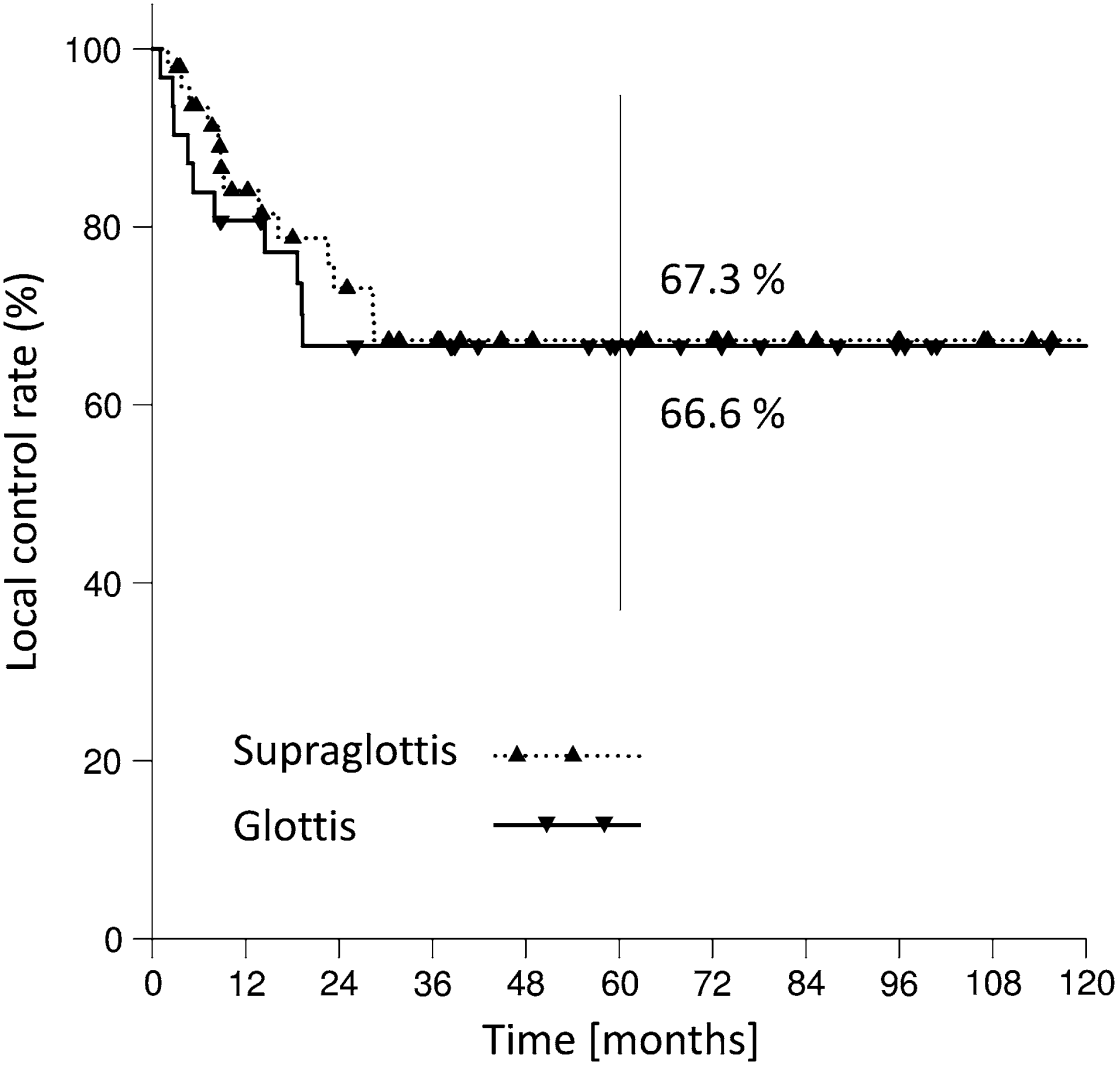
Glottic versus supraglottic laryngeal cancer. Local control rate for 79 patients with T4 primary tumor

These absolute figures translate into 2- and 5-year local control Kaplan–Meier estimates for TLM in T4a laryngeal cancer of 70.5 and 67.2 %, respectively. Calculated for glottic and supraglottic laryngeal cancer separately, the 5-year local control was 66.6, and 67.3 %, respectively. Local control rates for glottic and supraglottic carcinomas are given in Fig. 1.

Sixteen patients underwent total laryngectomy, due to oncological (*n* = 14) or functional reasons (*n* = 2) calculating 5-year organ preservation rate of 80 %.

### Second primary tumors

Second primary tumors were observed in 10 patients (16 %). In six of these patients, the second primary tumor was localized in the head and neck region; in four patients second primary occurred in the lung (*n* = 2), GI-tract (*n* = 2). The second primary tumors were diagnosed between 6 and 122 months (mean 36 months) after laser resection.

### Survival

**Fig. 2:**
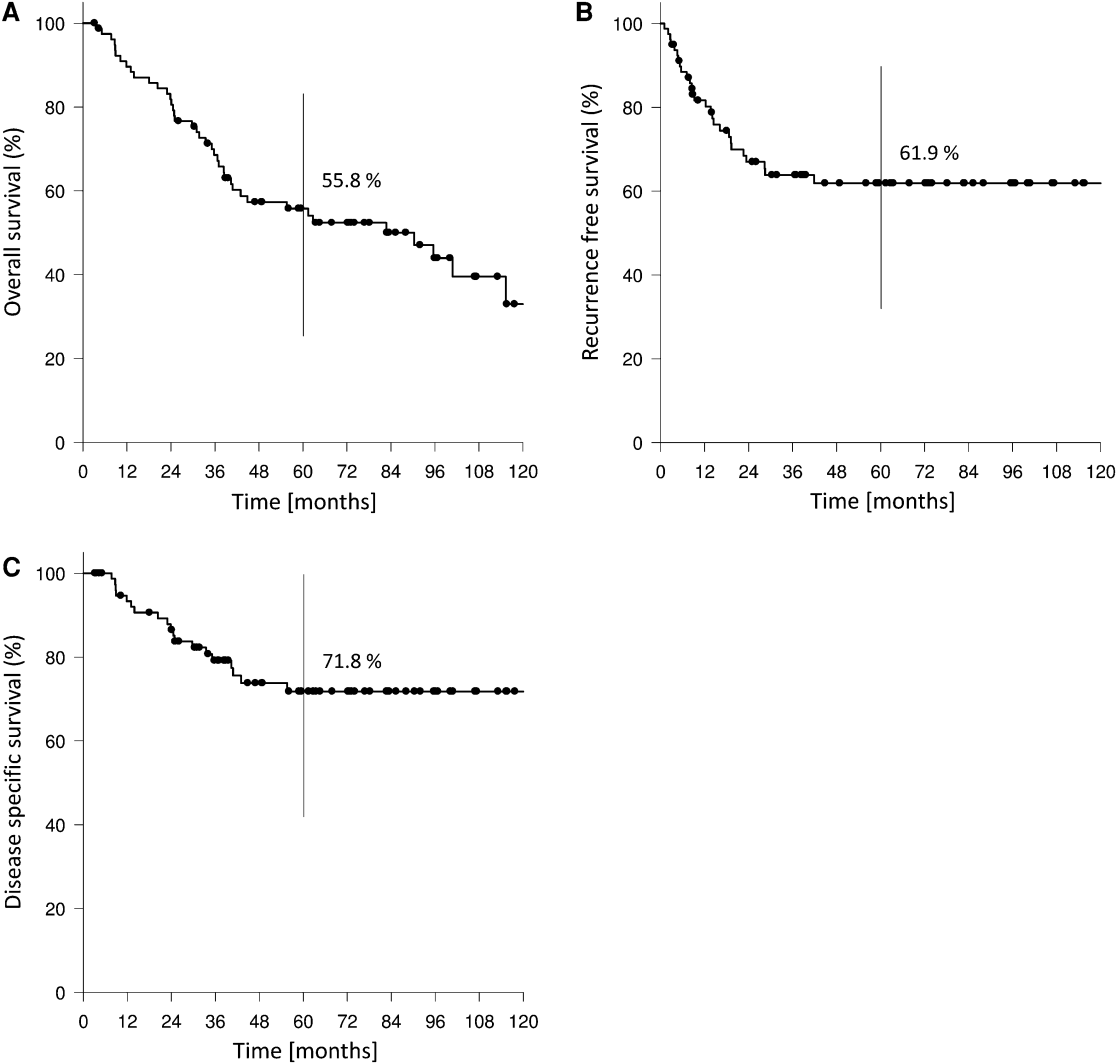
79 patients with T4 laryngeal cancer. **a** Overall survival rate. **b** Recurrence-free survival rate. **c** Disease-specific survival rate

**Fig. 3:**
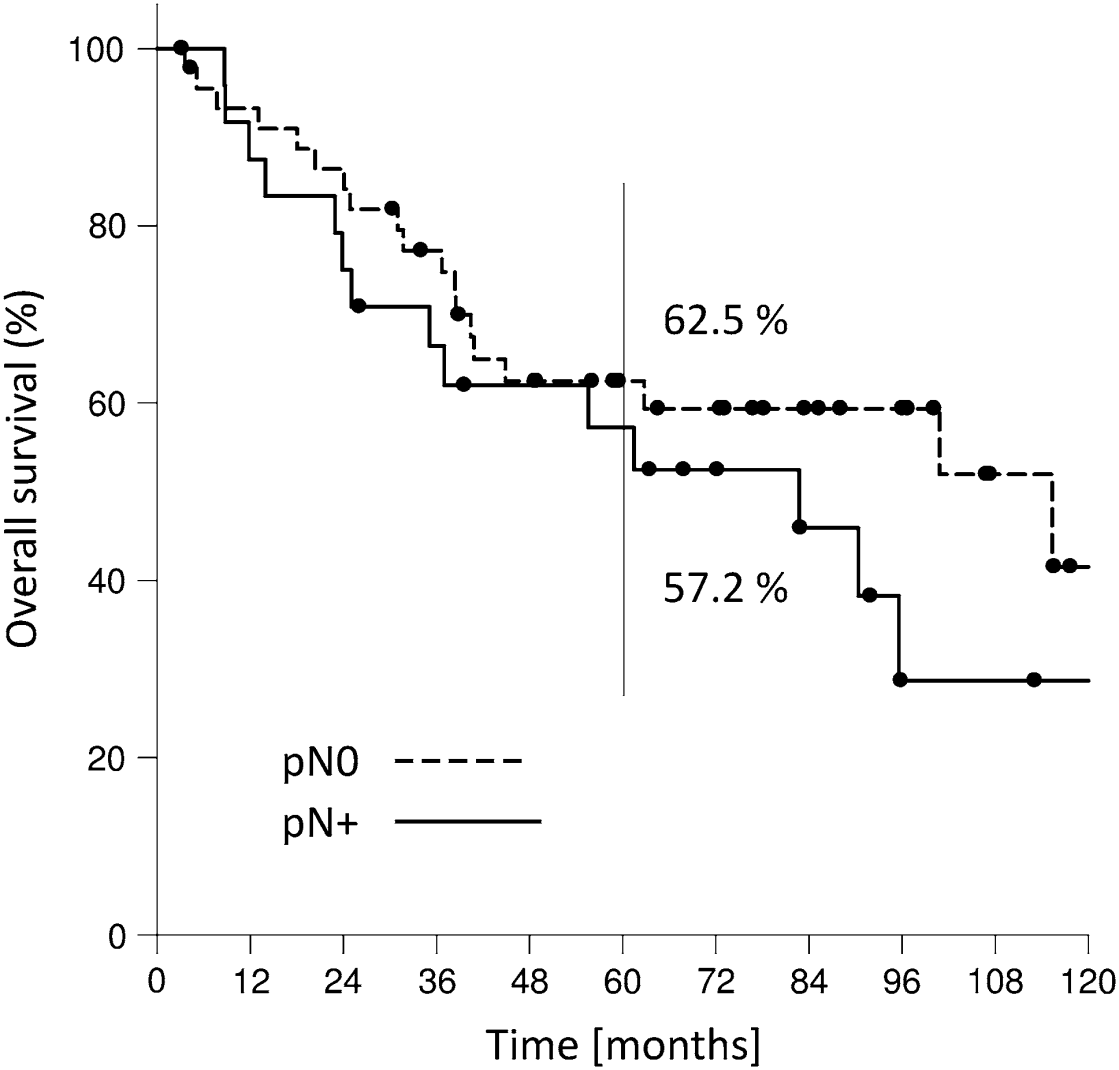
pN0 versus pN+. Overall survival rate for 79 patients with T4 laryngeal cancer

**Fig. 4:**
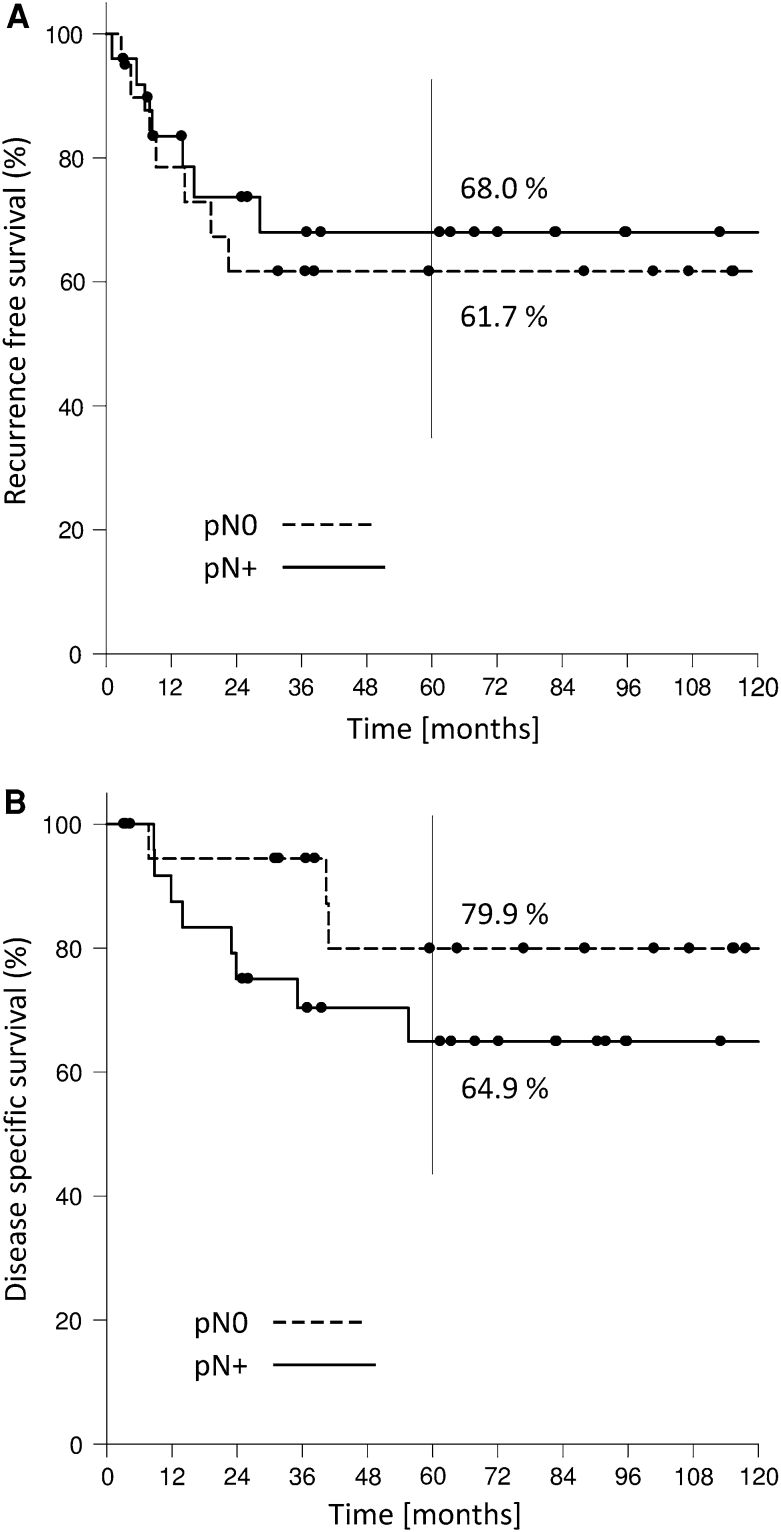
pN0 versus pN+ for 79 patients with T4 laryngeal cancer. **a** Recurrence-free survival rate. **b** Disease-specific survival rate

Kaplan–Meier estimates for 2- and 5-year overall survival, recurrence-free and disease-specific survival were 81.8 and 55.8%, 67.0 and 61.9 % and 86.5 and 71.8 %, respectively (Fig. 2a, b, c). If regarding glottic and supraglottic laryngeal carcinomas 5-year overall, recurrence-free and disease-specific survival were 65.3, 62.2, 75.4 % and 49.9, 61.6, 69.2 %. If pN-status was taken into account, the 2- and 5-year overall survival rate for pN0 patients were 86.4 and 62.5 % and 75.0 and 57.2 % for pN-positive patients (Fig. 3). The same effect was observable when evaluating the 2- and 5-year recurrence-free and disease-specific survival: 2- and 5-year recurrence-free survival for pN0 patients were 61.7 % each; for pN-positive patients it was 73.7 and 68.0 %. 2- and 5-year disease-specific survival for pN0 patients were 94.4 and 79.9 % and 75.0 and 64.9 % for pN-positive patients (Fig. 4a, b). A total of 41 patients (52 %) died. 19 (46 %) of these 41 patients died of TNM-related disease, 7 (17 %) died because of second primaries and 15 of 41 (37 %) intercurrently.

### Adjuvant (chemo)radiotherapy, recurrence rate and salvage treatment

Of 31 patients with glottic cancer, 12 received adjuvant (chemo)radiotherapy. Eight of 19 patients without adjuvant treatment developed tumor recurrence after an average timespan of 11.9 months. Six patients had local recurrence only. Three of them received local salvage surgery, one patient salvage laryngectomy and two patients local salvage surgery with adjuvant treatment. One patient with loco-regional recurrence received salvage laryngectomy. Four of the seven patients with salvage treatment died from the disease within an average of 20.9 months after recurrence. Three patients after salvage treatment were still alive without tumor after 42.1 months. One patient with regional and distant recurrence received palliative treatment only.

Five of 12 patients with glottic cancer and adjuvant treatment developed recurrence after an average of 12.2 months. One patient with local recurrence was treated with salvage laryngectomy and was still alive after a follow-up period of more than 72 months. Another patient with local recurrence died from disease-related causes 11 months after salvage laryngectomy. Three patients received palliative care due to distant or extensive local and regional recurrence.

After transoral laser microsurgery for supraglottic tumors (*n* = 48), 19 patients received adjuvant therapy. Twelve of 29 patients without adjuvant treatment developed tumor recurrence after an average of 8.8 months. Local recurrence occurred in nine patients and was treated by local salvage surgery in two, by salvage laryngectomy in six and by radiation alone in one case. Salvage treatment was successful in five patients, who were free of disease for an average follow-up period of 52.4 months. One patient with extensive nodal recurrence was treated by radiation only and died in the aftermath of a further regional and distant recurrence 29 months later. Two patients with distant metastases 3 and 5 months after primary treatment received palliative care only and passed away several months later.

Six of 19 patients with supraglottic cancer after TLM and adjuvant therapy developed recurrence. Three cases were local only and treated by local salvage surgery in 1 occasion and salvage laryngectomy in two. One patient was still free from tumor 62 months after salvage treatment, two died from disease-specific causes after 14 and 29 months, respectively. Three patients were treated with palliative care only due to distant metastases or extensive local and regional recurrence and survived for an average of 11.2 months.

### Postoperative complications and functional results

Postoperative laryngeal bleeding occurred in 2 out of 31 patients presenting with glottic carcinoma (6 %) and in 5 of 48 supraglottic (10 %) cases requiring coagulation and/or clipping in the operating room under general anesthesia.

Four patients with glottic carcinoma (8 %) needed a temporary tracheotomy, two intraoperatively at the end of the resection of the primary tumor as a prophylactic measure, one due to postoperative bleeding, and one due to laryngeal edema. In the supraglottic group, 9 of 48 (19 %) patients underwent a tracheotomy temporary (*n* = 5) or permanent (*n* = 4): five intraoperatively at the end of the surgical procedure, one because of persistent postoperative edema, one because of postradiogenic swelling of soft tissue and two because of persistent aspiration. One patient suffered a cardiac arrest during surgery that was solved successfully by the anesthesiologist.

Two patients (3 %) out of 79 (all with supraglottic cancer) needed total laryngectomy because of functional reasons and another two patients showed therapy related pneumonia due of aspiration.

Twenty-two of 31 patients (71 %) in the glottic carcinoma group needed temporary placement of a nasogastric feeding tube, which was removed in all but one case within 2 weeks. No gastrostomy tube was needed.

Forty out of 48 patients (83 %) with supraglottic cancer required a nasogastric feeding tube, which was removed within 4 weeks in 30 cases (73 %). In four cases (8 %) a permanent gastrostomy tube had to be placed.

## Discussion

The use of transoral laser microsurgery has been internationally accepted as a valid treatment option for early laryngeal cancer, however, its use in advanced laryngeal disease remains still controversial. In our series, we observed a 5-year local control of 67.2 % for pT4a laryngeal tumors. The 5-year larynx preservation rate was 80 %, 5-year overall, recurrence-free, and disease-specific survival rate was 55.8, 61.9, 71.8 %. Nonetheless, any discussion of oncologic outcomes is challenging and can be descriptive only, since evaluation of the evidence supporting the effectiveness of one treatment over the other is complicated by different stages, outcome measures, use of statistics and investigation of different laryngeal sites (supraglottic vs. glottic vs. all sites).

Total laryngectomy as primary or salvage therapy, different regimens of (chemo)radiotherapy and—in highly selected cases—open partial laryngectomy are the principal and widely used standard therapeutic options in many countries. Unfortunately, despite new therapeutic strategies like neoadjuvant chemotherapy and improved concomitant (chemo)radiotherapy procedures, survival rates have not improved within the last decades. Hoffman et al. [11] found decreased survival rates among patients with laryngeal cancer during the past two decades in the United States. In a large review of the National Cancer Data Base, the authors investigated data of 158,426 patients with laryngeal cancer. During this time there has been an increase in the non-surgical treatment of laryngeal cancer. Especially for advanced laryngeal cancer, 5-year observed and relative survival showed the best outcome for patients whose initial management was surgery either alone or combined with irradiation.

Few data have been published concerning primary surgical therapy in T4 laryngeal cancer. Ampil et al. [12] reported on 28 patients with T4 laryngeal cancer treated by total laryngectomy and postoperative radiotherapy. Kada et al. [13] investigated 33 patients with advanced T3/T4 laryngeal squamous cell carcinoma after total laryngectomy. The 5-year overall survival rates range between 24 and 43 %. Even though we solely present T4a carcinoma in our series, we observed a 5-year overall survival for glottic and supraglottic disease of 65.3 and 49.9 % being in favor for TLM in treatment of advanced laryngeal carcinomas. Therefore, the results achieved by TLM are comparable to the outcome after conventional total laryngectomy while having the advantage of organ preservation.

Primary (chemo)radiotherapy is associated with a high rate of failure and the necessity of salvage therapy (mostly by total laryngectomy). Different studies reported results in combination with neck dissection and total laryngectomy as salvage treatment [14–16]. Local and loco-regional control rates range from 43 to 65 %, disease-free survival rates are reported between 40 and 68 %. Nonetheless, total laryngectomy was necessary in up to 30 % of the cases. Patel et al. [17] compared primary (chemo)radiotherapy with total laryngectomy in the treatment of T4 laryngeal carcinoma. Of 34 included patients, 21 completed (chemo)radiotherapy and 13 underwent total laryngectomy with postoperative (chemo)radiotherapy. After (chemo)radiation 29 % revealed persistent/recurrent disease at the primary site whereas no local recurrence occurred after total laryngectomy. The authors conclude that although there was a high initial complete response rate after primary (chemo)radiotherapy, this response was not durable showing a high local recurrence rate within 1 year.

The therapeutic concept of neoadjuvant chemotherapy followed by radiation or surgery is still under clinical evaluation [18–20]. Forastiere et al. [21] randomly assigned 518 patients with locally advanced cancer of the larynx to one of three treatments: induction of cisplatin plus fluorouracil followed by radiotherapy, radiotherapy with concurrent administration of cisplatin, or radiotherapy alone. Eligibility for this study was based on the assertion that alternative surgical treatment would be total laryngectomy (stages III and IV). This makes a direct comparison difficult, as there were many T2- and T3-category tumors included that could have been treated by organ preserving surgery (TLM/open). Moreover, high-volume T4 carcinomas were excluded. Considering that the Forastiere series is a positive selection compared to our study, our 2-year laryngeal preservation of 86 % compares favorably to 75 % in the induction cisplatin with radiotherapy arm, 88 % in the concurrent chemoradiotherapy arm and 70 % in the radiotherapy alone arm. Also the 2-year local control rate of 71 % in the present study compared to 61, 78 and 56 % is in favor of TLM. Five-year estimates of the Forastiere study [22] show a laryngectomy-free survival of only 46.6 %, a loco-regional control of 68.6 %, an overall survival rate of 55 %, and a disease-free survival of 39 %. Compared to TLM, these 5-year results are not convincing and therefore induction chemotherapy followed by radiation cannot be recommended for locally advanced laryngeal carcinomas.

Regarding the concept of function preserving TLM for cancer of the upper aerodigestive tract, the surgical, oncological and functional principles do not differ from those that are used in open surgical procedures. Complete resection of the tumor is the main goal. However, preserving healthy tissue and the anatomical integrity of the larynx is an important benefit of TLM that is followed by an improved quality of life. The technical limits of TLM are reached when the tumor extents to the large vessels, or if severe postoperative functional problems are to be expected, mainly due to permanent aspiration.

After gaining experience with early stage cancers, the indication for TLM has gradually been extended to T4 laryngeal tumors by the senior author in the early 1980s [5]. Due to this expertise, numerous patients of the present series were referred to our hospital with the hope of organ preservation after being recommended total laryngectomy elsewhere. Therefore, a selective referral of very advanced tumors occurred and has to be considered when regarding the oncologic results of our institution.

Iro et al. [23] reported the oncologic results of transoral carbon dioxide laser microsurgery for 141 patients with supraglottic carcinomas, thereof 33 presented with T4 carcinoma (all sites). Five-year recurrence-free survival rates were 74.2 % for stage III and 45.3 % for stage IV. Hinni et al. [24] treated advanced glottic and supraglottic laryngeal cancer in 117 patients presenting with T2–T4 tumor, of these 33 patients with T4 carcinoma. Nineteen patients received TLM alone with or without neck dissection, 14 additionally had adjuvant (chemo)radiotherapy. Data for T4 patients only is not given. 5-year Kaplan–Meier estimates for all patients were local control 74 %; loco-regional control 68 %; disease-free survival 58 %; and overall survival 55 %. 5-year laryngeal preservation rate was 86 %. Rudert et al. [25]. investigated patients with advanced supraglottic laryngeal carcinoma which were treated with TLM. The achieved overall survival for T3 and T4 tumors was 47 %, however, data for T4 tumors only are not given.

The results achieved by TLM are comparable to the outcome after conventional total laryngectomy and superior to primary (chemo)radiotherapy. TLM with or without radiotherapy therefore is a valid treatment strategy for organ preservation. Furthermore, low morbidity and mortality and excellent oncologic and functional outcomes corroborate the importance of TLM as an attractive therapeutic option in T4a laryngeal cancer.

When reviewing the published data on complications and function, TLM shows advantages and validity as treatment option. Postoperative bleeding requiring microlaryngoscopic treatment occurred in seven cases (9 %) and one patient survived a cardiac complication intraoperatively. Total rates of severe toxic effects reported by Forastiere et al. [21] were 61 % (radiotherapy alone) and 81–82 % in the two arms associated with chemotherapy. In addition, the rate of patients being restricted to soft or liquid food because of swallowing difficulties ranged from 9 to 23 % after 1 year, depending on the therapy arm, and 14–16 % after 2 years. It is not reported how many patients required a temporary or permanent feeding tube. These figures are significantly worse than those reported for laser surgery in the present study or by other authors [23, 24] using TLM.

Regarding swallowing function, patients who undergo TLM for cancer of the hypopharynx and larynx usually have a good recovery of deglutition [26], which compares favorably to open partial resection or (chemo)radiotherapy. Even in our series of locally very advanced laryngeal carcinoma, the functional results are good. Two patients (3 %) required total laryngectomy because of aspiration, 2 (3 %) were treated for aspiration associated pneumonia and 4 (8 %) required a permanent gastrostomy or nasogastric feeding tube. Furthermore, speech intelligibility after laser microsurgery seems to be favorable when compared to total laryngectomy even with voice prosthesis [27].

## Conclusion

In selected patients with T4a squamous cell carcinoma of the larynx TLM with or without radiotherapy can be a valid option for organ preserving surgery. When compared to other surgical and non-surgical treatment options, the rates of organ and function preservation are very good and can be achieved without compromising the oncological results and with relatively low morbidity. Further benefits are the possibility to integrate transoral laser surgery into any therapeutic concept and the lack of necessary reconstructive surgery. These results are very satisfactory and encouraging to continue and recommend TLM for advanced laryngeal carcinoma, but as it is the data of only one institution, results should be validated by prospective multicenter studies.
